# Immune Metabolism–An Opportunity to Better Understand Allergic Pathology and Improve Treatment of Allergic Diseases?

**DOI:** 10.3389/falgy.2022.825931

**Published:** 2022-03-09

**Authors:** Alexandra Goretzki, Jennifer Zimmermann, Yen-Ju Lin, Stefan Schülke

**Affiliations:** Molecular Allergology, Paul-Ehrlich-Institut, Langen, Germany

**Keywords:** immune metabolism, allergy, immune effector molecules, metabolic state, Warburg

## Introduction

Metabolism is defined as “*the sum of the chemical reactions that take place within each cell of a living organism and that provide energy for vital processes and for synthesizing new organic material*.” (Britannica, https://www.britannica.com/science/metabolism accessed 22.09.21).

Recent high-level publications have shown the metabolism of immune cells and their effector function to be closely connected. To better understand these connections between immune system activation and metabolic states is the aim of the newly formed research field called “immune metabolism”.

**Figure 1:**
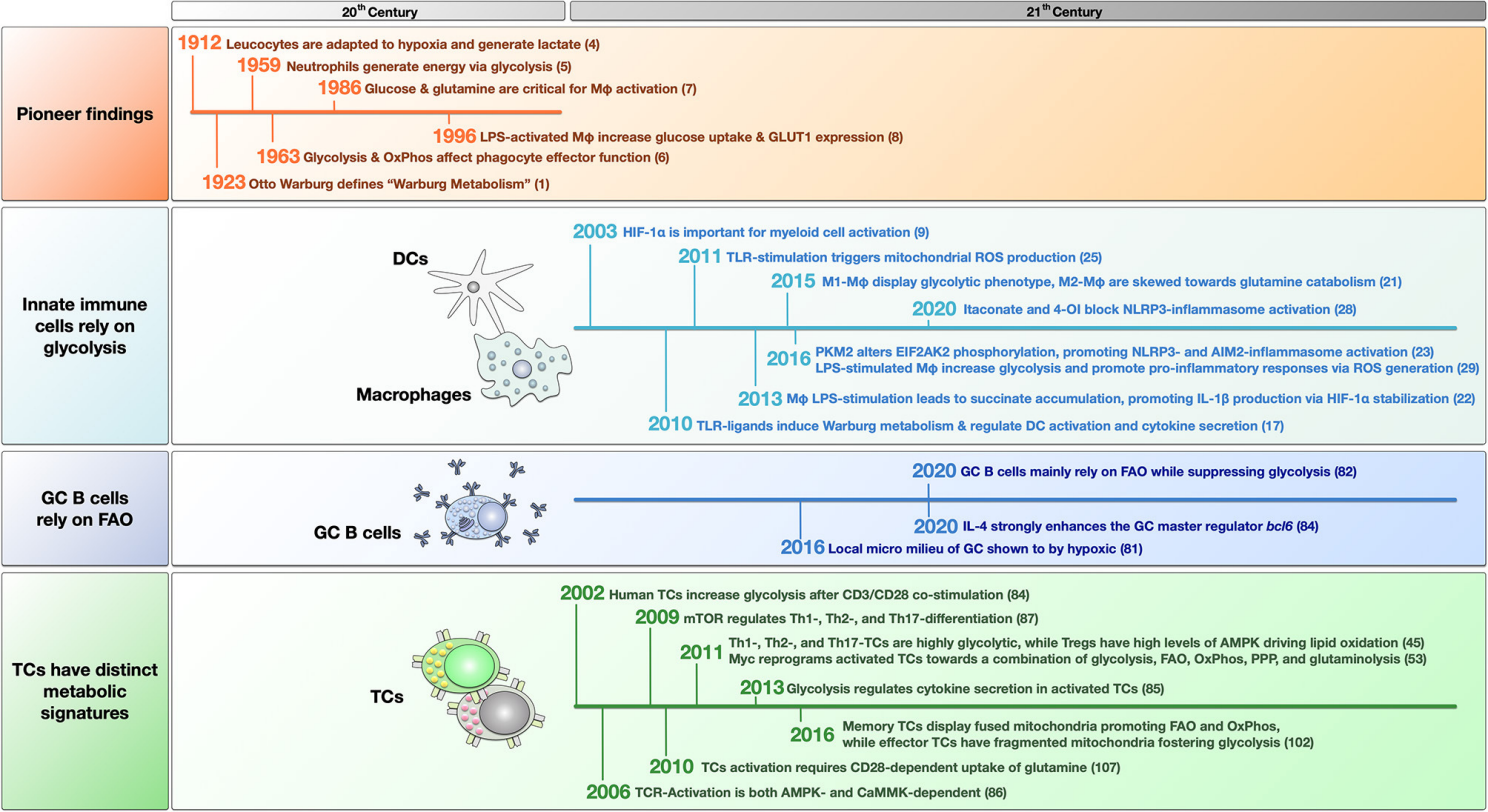
Timeline of pioneering and breakthrough papers that have helped form the field of immune metabolism. The findings are grouped into pioneer studies in the last century, and recent findings about innate immune cells, germinal center (GC) B cells, and T cells. For more detailed information see the main text. LPS, lipopolysaccharide; OxPhos, oxidative phosphorylation; FAO, fatty acid oxidation; GLUT1, glucose transporter 1; HIF-1α, hypoxia-inducible factor 1 alpha; TLR, “Toll” like receptor; ROS, reactive oxygen species; Mφ, macrophage; 4-OI, 4-octyl itaconate; PKM2, pyruvate kinase isozyme M2; EIF2AK2, eukaryotic translation initiation factor 2 alpha kinase 2; TC, T cell; mTOR, mammalian target of rapamycin; PPP, pentose phosphate pathway; AMPK, adenosine monophosphate-activated protein kinase; CaMMK, Ca^2+^-calmodulin-dependent protein kinase kinase; TCR, T cell receptor.

This opinion article will summarize some of the groundbreaking studies that established the field of immune metabolism and highlight pioneering metabolic studies that increased our understanding of allergic pathology and introduced potential treatment targets. A timeline of the discussed studies is provided in Figure 1.

## A Short History of Immune Metabolism

### Cancer Cells Predominantly Produce Their Energy by Generating Lactate From Glucose

In 1923, the German biochemist Otto Warburg (1) was the first scientist to describe metabolic changes in cancer cells. He observed cancer cells to preferentially produce lactate from glucose even under conditions of sufficient oxygen supply, which was termed aerobic glycolysis (1).

Based on his findings, Warburg (2) hypothesized that cancer development results from cumulative low-level respiratory injury resulting in an irreversible metabolic derangement, which we now know as mitochondrial damage. While many cells die due to the obtained respiratory damage, some cancer cells replace mitochondrial respiration with glucose fermentation, resulting in the observed aerobic glycolysis (2).

What is now the relevance of his findings? Under normal circumstances, cells take up glucose from their surroundings and metabolize the C6-body in a cytoplasmic process termed glycolysis into two molecules of pyruvate (C3-body). Pyruvate can then be transported in the mitochondrion, where it is used in the Krebs cycle to generate energy in the form of reduction equivalents.

However, in cancer cells, highly active cells, and activated immune cells, pyruvate can also be metabolized into lactate which is subsequently exported from the cell, termed “Warburg metabolism” (3).

Energetically, fully metabolizing glucose in the mitochondrion generates 36 molecules of ATP per molecule of glucose, while Warburg metabolism only generates 2 molecules ATP/ molecule of glucose. Therefore, Warburg metabolism is energetically wasteful but can be sustained under oxygen-deprived or -free conditions (commonly found in acutely inflamed/infected tissues). Furthermore, it is faster and allows activated immune cells to rapidly fulfill the increased energy demands arising upon acute activation.

### Metabolism Contributes to Immune Cell Effector Function

After Warburg's revolutionary findings, it took some decades until scientists realized that metabolic changes are also relevant for immune cell function. Although Levene and Meyer (4) described in 1912 that leucocytes might be adapted to hypoxia and exhibit high levels of lactate production, it lasted until 1959 for Sbarra and Karnovsky (5) to show neutrophils (which inherently have very few mitochondria) to generate their energy via glycolysis both under aerobic and anaerobic conditions.

In another pioneering study in 1963, Oren et al. (6) analyzed three different types of macrophages isolated from guinea pigs. In this early study, alveolar macrophages were shown to switch to oxidative phosphorylation (OxPhos) in the mitochondrion to fuel phagocytosis, while peritoneal exudate monocytes and polymorphonuclear leukocytes were more dependent on glycolysis (6). In their hands, the respiratory activity of alveolar macrophages was three to ten times greater than that of the other two types of phagocytes (6). Consequently, inhibition of either aerobic metabolism or OxPhos suppressed particle uptake in alveolar macrophages (6).

**Figure 2:**
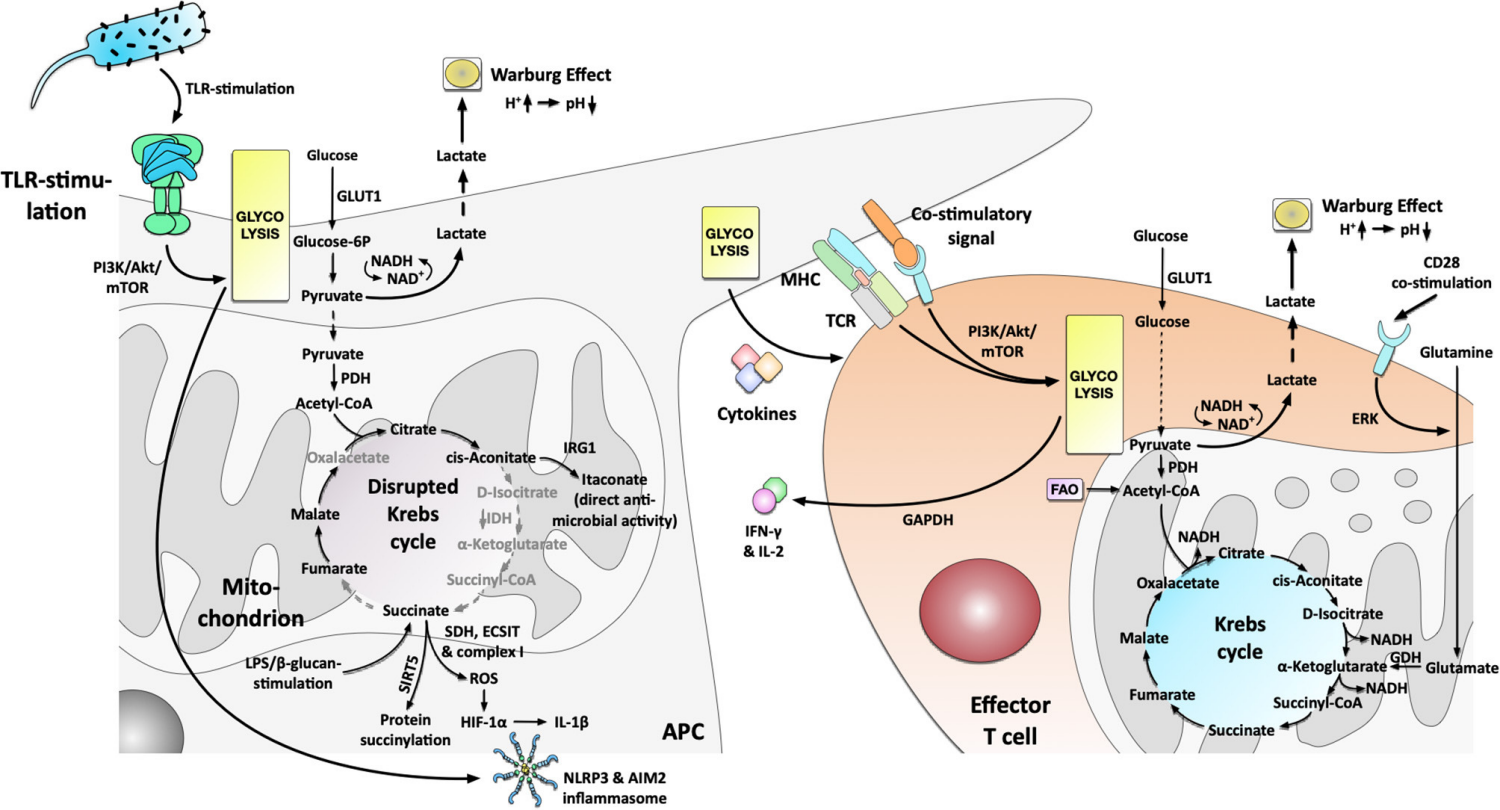
Metabolic phenotype and the connection to immune cell effector function in activated Antigen Presenting Cells and effector T cells. Antigen Presenting Cells (APCs) activated by e.g., TLR-stimulation switch to a predominant production of energy via glycolysis that drives cytokine production and results in the production and excretion of lactate (Warburg Effect). The switch toward glycolysis is driven by the PI3K/Akt/mTOR-axis. The disrupted Krebs cycle resulting from an undersupply of pyruvate in the mitochondrion is used to (I) generate important immune effector molecules (e.g., itaconate, ROS), (II) fuel post-translational protein modification (e.g., succinylation or acetylation), and (III) free up the mitochondria from energy production in order to promote inflammatory responses via an SDH-, ECSIT- and complex I-dependent production of ROS which in turn drive HIF-1α- and inflammasome-dependent IL-1β production. Activated T cells PI3K/Akt/mTOR-dependently increase their glycolysis but also retain metabolic flexibility with the ability to fuel the mitochondrion with glutamine. Increased glutamine uptake and metabolism is regulated by CD28-dependent ERK-signaling. Moreover, the glycolytic enzyme GAPDH regulated IFN-γ secretion by interacting with IFN-γ mRNA. For more information see text. TLR, “Toll”-like receptor; GLUT1, glucose transporter 1; PDH, pyruvate dehydrogenase; IRG-1, immune-responsive gene 1; IDH, isocitrate dehydrogenase; SDH, succinate dehydrogenase; ROS, reactive oxygen species; HIF-1α, hypoxia inducible factor 1 alpha; SIRT5, Sirtuin 5; ECSIT, evolutionarily conserved signaling intermediate in Toll pathways; MHC, major histocompatibility complex; TCR, T cell receptor; PI3K, phosphoinositide 3-kinase; Akt, protein kinase B; mTOR, mammalian target of rapamycin; GAPDH, glyceraldehyde 3-phosphate dehydrogenase; GDH, glutamate dehydrogenase; ERK, extracellular regulated kinase; FAO, fatty acid oxidase.

This study firstly hinted at a connection between immune cell effector function and changes in their metabolic phenotype. Toward the end of the century, the advent of modern analysis methods allowed for more detailed analyses of the involved mechanisms and intracellular molecules: In a key study on macrophage metabolism, published in 1986 by Newsholme et al. (7), the authors demonstrated the increase of both glycolysis-related enzymes (e.g., glucose-6-phosphate dehydrogenase and 6-phosphogluconate dehydrogenase) and glutaminase activity upon activation of thioglycollate-elicited mouse peritoneal macrophages, showing both glucose and glutamine to be critically important for macrophage activation. In line with these results, Fukuzumi et al. (8) reported in 1996 that lipopolysaccharide (LPS)-activated macrophages increased their glucose uptake, linked to a higher expression of glucose transporter 1 (GLUT1, Figure 2).

## Immune Metabolism in the Current Century

### Glycolysis Is One of the Major Metabolic Pathways for Innate Immune Cells

A milestone was set. With the results of Newsholme and Fukuzumi, the intracellular signal pathways and their connection to cell metabolism became the focus of immune metabolic research in the 21^st^ century. In this section, we sum up the recent breakthrough findings in immune metabolism.

In 2003, the study of Cramer et al. (9) provided an important molecular link between oxygen deprivation in inflamed tissues and immune cell function. Here, the hypoxia-inducible factor 1α (HIF-1α) was shown to be critically important for myeloid cell activation (9).

Under hypoxic conditions, HIF-1α drives the expression of many genes but is rapidly degraded by the ubiquitin-proteasome pathway under ambient conditions due to its oxygen sensitivity (10). Conditional knock-out of HIF-1α drastically reduced the cellular ATP pool, myeloid cell aggregation, cell motility, invasiveness, and bacterial killing in isolated peritoneal macrophages, and suppressed inflammation in several *in vivo* models of acute and chronic inflammation. These wide-ranging effects demonstrate the importance of HIF-1α in regulating both myeloid cell survival and effector function (9). Here, HIF-1α was shown to critically regulate pathways essential for maintaining energy homeostasis in myeloid cells (9).

In line with these results, low oxygen levels have been reported at sites of acute inflammation (11–16). Therefore, the observed HIF-1α-dependent regulation of immune cell function likely adapts innate immune cells to the hypoxic conditions under which they are required to function.

In 2010 Krawczyk et al. (17) provided further evidence that a switch toward increased glycolysis is a hallmark of dendritic cell (DC) activation: Stimulation of mouse DCs with different “Toll”-like receptor (TLR)-ligands (LPS, CpG, and heat-killed bacteria) resulted in a transition from OxPhos to aerobic glycolysis (Warburg metabolism, Figure 2), which regulated DC activation and cytokine secretion (17). This metabolic switch depended on the phosphatidyl inositol 3-kinase (PI3K)/ protein kinase B (Akt) pathway while being antagonized by both adenosine monophosphate (AMP)–activated protein kinase (AMPK, regulating OxPhos) and the anti-inflammatory cytokine IL-10 (17). AMPK is a serine/threonine kinase that regulates metabolic processes and maintains energy homeostasis [reviewed in (18)]. Hawley et al. (19) reported in 2005 that liver kinase B1 (LKB1) and calcium/calmodulin-dependent protein kinase kinase β (CaMKKβ) are important upstream regulators for AMPK.

Therefore, these groundbreaking results highlighted two things: (I) activated TLRs induce signaling events classical associated with immune cell activation, and (II) the activated TLRs change cellular metabolic pathways in order to sustain both the energy levels and metabolic resources which the activated cells require to support the TLR-induced changes in gene expression (17).

Besides DCs, M1 macrophages also strongly rely on glycolysis (20). Using a combined experimental and computational pipeline, Jha et al. (21) confirmed M1 macrophages to display a mainly glycolytic phenotype while M2 macrophages were skewed toward glutamine catabolism. Moreover, Jha et al. (21, 22) demonstrated that LPS-stimulation disrupts the Krebs cycle, breaking it at two points after citrate (resulting in itaconate synthesis) and after succinate (resulting in pro-inflammatory IL-1β production and protein succinylation, Figure 2).

Xie et al. (23) reported in 2016 that pyruvate kinase isozyme M2 (PKM2), the enzyme catalyzing the rate-limiting step of glycolysis, altered eukaryotic translation initiation factor 2 alpha kinase 2 (EIF2AK2) phosphorylation, and further promoted NLRP3- and AIM2-inflammasome activation in macrophages (Figure 2). Interestingly, the PKM2 inhibitors shikonin and C16 could protect mice from lethal endotoxemia and polymicrobial sepsis, showing that targeting metabolic pathways might allow for new treatment options (23).

Since increased glycolysis was first found in cancer cells, targeting glycolysis as treatment option in cancer therapy has been tested and discussed (24). Also targeting glycolytic innate immune cells in allergy could be a future attractive therapeutic option. For instance, blocking the activation of either dendritic cells or macrophages might interfere with the antigen (allergen) presentation process. However, here more detailed studies are needed. A major challenge in the future will be to link the reported *in vitro* results with future *in vivo* findings, improve the specificity when targeting different cell types, and investigating the long-term safety of targeting glycolysis.

### The Combination of Increased Glycolysis and a Broken Krebs Cycle Is Elegantly Used in Activated Immune Cells to Form Important Immune Effector Molecules

In another high-ranking study in 2011, West et al. (25) reported TLR-stimulation to trigger mitochondrial reactive oxygen species (ROS) production with bactericidal activity. Stimulation of mouse macrophages with ligands for cell surface TLRs (TLR1, TLR2, TLR4), but not ligands for endosomal TLRs (TLR3, TLR7, TLR8, TLR9), triggered mitochondrial ROS production (25). Mechanistically, tumor necrosis factor receptor-associated factor 6 (TRAF6)-dependent ubiquitinylation of evolutionarily conserved signaling intermediate in Toll pathways (ECSIT) was shown to drive complex I-dependent ROS production (Figure 2) (25).

In a fascinating study, Tannahill et al. (22) also reported a critical function of mitochondrial metabolites in the induction and regulation of macrophage responses. Here LPS-stimulation resulted in glycolytic activity and Warburg metabolism (as described above) (22). Furthermore, they found that LPS-stimulation also led to the accumulation of the Krebs cycle intermediate succinate via glutamine-dependent anaplerosis and the “GABA-shunt” pathway (22). Here, succinate acted as a cell-intrinsic danger signal driving pro-inflammatory IL-1β production via stabilization of HIF-1α (termed “pseudohypoxia,” Figure 2) (22, 26). Moreover, succinate was used as a substrate to post-translationally modify certain target proteins via Sirtuin 5 (SIRT5)-dependent succinylation (Figure 2) (22), thereby further contributing to immune effector function.

In line with this, the disrupted Krebs cycle can also be used to produce other molecules with either direct or indirect anti-microbial and immune-modulating activity like ROS, nitric oxide species (NOS), or the natural antibiotic molecule itaconate (from citrate-derived cis-aconitate via immune-responsive gene 1 (IRG-1), Figure 2) [reviewed in (27)]. For example, a recent study showed that Krebs cycle-derived itaconate and its derivative 4-octyl itaconate (4-OI) could block NLRP3-inflammasome activation (28). Hooftman et al. (28) found that 4-OI dicarboxypropylated NLRP3 at position C548, blocking the interaction between NLRP3 and NIMA-related kinase 7 (NEK7) upon LPS/ATP/Nigericin-stimulation. Besides, 4-OI also inhibited NLRP3-dependent IL-1β release from PBMCs and reduced inflammation in a model of urate-induced peritonitis *in vivo* (28).

In a follow-up study, Mills et al. (29) elegantly showed that increased energy production via glycolysis (albeit being energetically wasteful) in LPS-stimulated macrophages frees up the cell's mitochondria from ATP synthesis in order to promote pro-inflammatory responses via the generation of ROS (driving HIF-1α stabilization and subsequent IL-1β production, Figure 2). This process was driven by a combination of increased mitochondrial oxidation of succinate via succinate dehydrogenase (SDH) and elevation of the mitochondrial membrane potential with subsequent generation of ROS via mitochondrial complex I (Figure 2) (29). Therefore, mitochondria act as signaling hubs that link macrophage activation, metabolic shift, and immune effector function via electron transfer in the electron transport chain (ETC). These results confirm and further explain the results of West et al. (25) that observed TLR-stimulation to drive ROS generation via a change in cellular metabolism (see above). In addition to other effects, like e.g., intracellular signaling as a second messenger or microbicidal activity, a highly increased production of endogenous ROS was also reported to be involved in allergic asthma pathogenesis (30, 31). Furthermore, the reactions of several allergy-related cell types to ROS (and reactive nitrogen species) during initiation of pulmonary allergy were reviewed in (32, 33). Interestingly, for example, mast cells exposed to ROS were activated and released both histamine and serotonin (33).

In addition to the activation of cells by ROS, the accessibility to anti-oxidants like glutathione (GSH) seems to play a role in developing Th2 responses: Already in 1998, antigen-presenting cells (APCs) were found to preferentially induce Th2 responses if GSH was depleted using e.g., ethanol, or diethyl maleate (34), which was confirmed in 2016 by Brundu et al. (35) using murine leukemia virus infection. Unfortunately, in these studies the immune metabolism was not investigated. Therefore, a further focus on anti-oxidative molecules, like glutathione and its intracellular mode of action, could be a promising strategy for preventing Th2 responses caused by ROS.

All these and many other studies in the meantime impressively demonstrated that the switch to mainly glycolytic metabolism and the “disrupted Krebs cycle” (resulting from an undersupply of pyruvate in the mitochondrion) (36) are efficiently used in immune cells, supplying metabolites which either directly or indirectly promote the respective cell's effector function (Figure 2) (36). Therefore, these cells have elegantly adapted their metabolism to efficiently function under the highly hypoxic conditions found in infected/inflamed tissues.

However, Lachmandas et al. (37) showed that not all TLR-ligands enhance glycolysis while suppressing OxPhos. Comparing the effects of the TLR2-ligand Pam_3_CysSK_4_ to the TLR4-ligand LPS on human CD14^+^ monocytes, they observed that only LPS-, but not Pam_3_CysSK_4_-stimulation resulted in a reduced rate of mitochondrial respiration while both TLR-ligands promoted a glycolytic phenotype (37). Indeed, levels of OxPhos increased upon stimulation with Pam_3_CysSK_4_, and OxPhos was shown to be relevant for Pam_3_CysSK_4_-triggered phagocytosis while both OxPhos and glycolysis contributed to cytokine production (37).

Therefore, metabolic programs induced by different stimuli considerably vary and have the potential to significantly contribute and shape the output of the respective immune cell and, consequently, the overall immune responses. These results are also of relevance in allergic responses and should be considered when developing novel therapies. Currently, TLR ligands are among the most attractive adjuvants for allergen immunotherapy. However, as described in this chapter, depending on the stimulus, they may trigger different outcomes, including either Th1- or Th2-biased immune responses. For example, a study showed that house dust mite (HDM) allergens could induce allergic rhinitis in the nasal mucosa via TLR2, but induction of allergic asthma was TLR4-dependent in the airway (38). Besides, HDM-induced TLR2/4 activation, both HDM allergens mediated ROS production and led to Th2 cytokine secretion, suggesting that TLR2- or TLR4-activation might not be the optimal strategy for adjuvants in HDM immunotherapy. Therefore, studying the underlying activation of immune metabolism induced by allergens with or without additional adjuvants should be taken into consideration during treatment development.

### Neutrophils and MDSCs Are Metabolically Flexible and Use Different Metabolic Pathways for Distinct Effector Functions

When looking at the connection between immune cell function and metabolism, the picture for neutrophils and MDSCs is less clear-cut. Both cell types display pronounced metabolic flexibility with different metabolic pathways contributing to distinct effector functions.

Neutrophils eliminate pathogens by different effector functions, including phagocytosis, production of ROS, the release of genomic DNA, such as neutrophil extracellular traps (NETs), and release of cytotoxic granules (39, 40).

Early work of Levene and Meyer suggested neutrophils to mainly rely on glycolysis [(4), see above]. Additionally, neutrophils were shown to contain only few mitochondria (41), as well as significantly lower levels of both oxidative phosphorylation complexes (42) and the mitochondrial enzymes glutamate dehydrogenase and fumarase (41). Accordingly, neutrophil phagocytotic function was shown to predominantly depend on glycolytic metabolism but not on mitochondrial respiration (5, 43).

However, in tumor microenvironments characterized by low glucose supply, neutrophils were found to adapt their metabolism to mitochondrial fatty acid oxidation in order to maintain NOX-2-dependent ROS production (44). Moreover, neutrophils were shown to switch to glutamine metabolism under glucose-limited, pathophysiological conditions (45, 46).

In line with these results, recent research has suggested that neutrophils utilize diverse metabolic pathways including glycolysis, glutaminolysis, the pentose phosphate pathway, fatty acid oxidation (FAO), and the Krebs cycle to perform distinct effector functions (47): Neutrophil chemotaxis was found to be impaired when zebrafish neutrophils either lack a functional membrane potential or ATP synthase (48), suggesting the importance of mitochondria during neutrophil transmigration into tissues.

Watts et al. (49) showed that under hypoxic conditions in a lung injury model, neutrophils scavenge proteins from their environment in order to use amino acids derived from lysosomal degradation of these proteins to maintain their central carbon metabolism. Therefore, neutrophils seem to be efficiently adapted to exploiting the protein-rich milieu in the inflamed lung (49).

In 2014 Rodriguez Espinosa et al. (50) showed neutrophil NET formation to be divided into two distinguishable metabolic phases: (I) chromatin decondensation being independent of exogenous glucose and (II) NET release strictly dependent on both exogenous glucose and glycolysis.

Finally, generation of cytosolic NOX-dependent, neutrophil-derived ROS for NET formation was shown to depend on either the production of NADPH by the glucose-dependent pentose phosphate pathway (51) or glutamine metabolism (52). Some studies have also suggested mitochondrial metabolism to contribute to ROS production via complex I and II (53, 54). Here, glucose metabolism seems to be required for early burst ROS production while fatty acid metabolism and mitochondrial function contribute to prolonged H_2_O_2_ production during late ROS production [reviewed in (47)].

Previous studies indicated that neutrophils play an important role during allergic sensitization to pollen and cat dander extracts (55–57). Neutrophils can affect allergic responses by degrading allergens, enhancing eosinophil migration, and, interestingly, producing (the previously discussed) ROS that facilitate allergic inflammation (55–57). However, currently, there is no study analyzing the metabolic changes of neutrophils in allergy, which could be an exciting future research topic.

Myeloid-derived suppressor cells (MDSCs) are a heterogeneous population of immature myeloid progenitor cells that share the ability to suppress adaptive immune responses in different pathological conditions, including cancer, infections, and autoimmune diseases (58–61). Mechanistically, the suppressive functions of MDSCs are based on (I) the depletion of L-arginine and L-cysteine, which are essential for sustaining T cell function, (II) secretion of ROS and NOS that disturb IL-2 and T cell receptor signaling, (III) release of immune suppressive cytokines such as IL-10 and TGF-β, and (IV) the expression of different immune checkpoint modulators (e.g., PD-L1) (62–64). Thereby MDSCs suppress T cell proliferation, NK cell cytotoxicity and survival, interfere with T cell homing, and promote the development of regulatory T cells (65, 66). In the tumor microenvironment MDSCs furthermore directly support tumor cells by promoting cancer stemness, angiogenesis, and metastasis formation (67, 68).

MDSCs comprise two populations: CD11b^+^Gr1^int^Ly6G^−^Ly6C^+^ monocytic (M-MDSC) and CD11b^+^Gr1^high^Ly6G^+^Ly6C^−^ granulocytic (G-MDSC) MDSCs (59, 69).

Metabolically, MDSCs are described as metabolically flexible, employing glycolysis, amino acid metabolism, and FAO for their suppressive function. One hallmark of MDSC metabolism is an increase in amino acid metabolism, which is used to deplete the amino acids arginine (via Arg1), tryptophan [via indoleamine 2, 3-dioxygenase (IDO)], and cysteine from the extracellular space, thereby preventing their uptake by T cells (70).

While M-MDSCs mainly rely on Warburg metabolism and high glutamine uptake (71) G-MDSCs seem to utilize both glycolysis and OxPhos for their energy production and suppressive function (72, 73). Jian et al. (72) recently reported both extracellular acidification rate (ECAR) and glycolytic enzymes to be upregulated in total MDSCs. Here, the two key glycolysis enzymes hexokinase, and glyceraldehyde-3-phosphate dehydrogenase (GAPDH), were shown to protect MDSCs from ROS-mediated apoptosis (72).

Recently, Hossain et al. (74) could show tumor-infiltrating human G-MDSCs from colon adenocarcinoma, clear cell kidney carcinoma, and breast ductal carcinoma to undergo metabolic reprogramming from glycolysis to FAO and OxPhos. The respective MDSCs were characterized by an increased mitochondrial mass, increased fatty acid uptake, upregulated expression of the fatty acid translocase CD36, and recombinant macrophage scavenger receptor 1, as well as enhanced levels of FAO cycle enzymes carnitine palmitoyltransferase 1 and 3- hydroxyacyl-CoA dehydrogenase, and an augmented oxygen consumption rate (74). Increased uptake of exogenous fatty acids by tumor MDSCs enhanced their immunosuppressive activity on T cells via the production of suppressive cytokines, thereby promoting tumor progression (74). Conversely, inhibition of FAO significantly delayed tumor growth in a T cell-dependent manner and enhanced the anti-tumor effect of adoptive T cell therapy, clearly demonstrating the importance of fatty acid metabolism for MDSC function (74).

In line with this, Al-Kami et al. (75) in 2017 demonstrated, the tumor-derived cytokines G-CSF and GM-CSF to STAT3- and STAT5-dependently induce the expression of lipid transport receptors, resulting in increased lipid uptake in the tumor microenvironment. Here, lipid uptake increased oxidative metabolism and promoted immunosuppressive mechanisms of MDSCs (75).

The currently available studies about MDSCs in allergy are mainly focused on asthma. As described above, targeting MDSCs is considered a possible cancer treatment, and this idea was also suggested for asthma therapy. However, so far the results are debatable: Some *in vivo* studies showed, that injecting MDSCs could suppress infiltration of inflammatory cells as well as allergen-specific Th2 cytokine and IgE levels in lung tissues (76, 77), making MDSCs an attractive treatment for asthma. In contrast, another study indicated that mast cells increased IgE-mediated IL-13 secretion when in contact with MDSCs in co-cultures, and *i.p*. injection of MDSC even aggravated airway hyper-responsiveness in mice (78). These controversial studies show, that more detailed analyses are required. A recent study in 2021 by Geffen et al. (79) demonstrated that prostaglandin E2 receptor 4 (EP4) agonist-primed MDSCs increased arginase-1- and nitric oxide synthase-2 production. Besides, the treatment with the EP4 agonist increased immunosuppressive activity of MDSCs on T cell responses in asthmatic mice (79). As prostaglandin E2 is one of the intermediates that occur during a disrupted Krebs cycle, the investigated increase of arginase-1/nitric oxide synthase-2 can probably be attributed to changes in cellular metabolism. Therefore, elucidating the detailed metabolic changes and either targeting or modulating MDSC metabolism in allergy could be a viable strategy for future therapy development.

## Energy Generation via OxPhos is More Relevant in Adaptive Immune Responses

### In Contrast to Other B Cell Subsets Germinal Center B Cells Mainly Rely on Fatty Acid Oxidation

Germinal centers (GC) are specialized micro environments in which antigen-activated B cells expressing high-affinity B cell receptors (BCRs) are positively selected to undergo clonal expansion, immunoglobulin class switching, and affinity maturation (80). All these processes are highly energy intensive. The local micro milieu of GC was shown to be hypoxic (81). Consequently, GC B cells were believed to energetically rely on oxygen-independent glycolysis. B cells employ both aerobic glycolysis and FAO, while GC B cells were recently shown to mainly rely on oxidation of both endogenous and exogenous fatty acids for energy generation while inherently suppressing glycolysis (82). Weisel et al. (82) speculated that this prominent reliance on FAO of GC B cells might be because these cells locally acquire their energy in the form of fat derived both from cell membranes and fat energy stores of cells dying within the germinal center.

Related to allergy, Haniuda et al. (83) showed IL-4 to strongly enhance expression of the master regulator of GC B cell differentiation, *Bcl6*. Interestingly, IL-4 stimulation did not increase lactate production, but enhanced mitochondrial membrane potential, suggesting a shift toward mitochondrial metabolism (83). During this IL-4-induced metabolic shift, α-ketoglutarate accumulated dependent on mitochondrial respiration, which was found to be required for the activation of the *Bcl6* via epigenetic changes (83). Besides this, additional information on metabolic changes in GC B cells in allergy is rare. However, IL-4 activation of B cells was recently reviewed (36). To address e.g., which metabolic pathways may contribute to IgE class-switching in GC B cells, could both contribute to better understanding allergic pathology and improve therapy development in the future.

### Effector and Regulatory T Cell Subsets Have Different Metabolic Signatures

In 2002, Frauwirth et al. (84) identified in human T cells (TCs) that CD3/CD28 co-stimulation PI3K- and Akt-dependently increases glycolic flux with enhanced GLUT1 expression, glucose uptake, and increased glycolysis with 90% of glucose being converted to lactate (Figure 2). However, TCs were also metabolically flexible with the ability to increase O_2_-consumption (suggesting increased OxPhos) by 4-fold under conditions of glucose limitation (84). Consequently, Frauwirth et al. (84) concluded, that the high production of lactate by the activated TCs is not due to their inability to increase OxPhos. Indeed, they were proven right as we now know, that the disrupted Krebs cycle is used to generate important immune effector molecules (see Immune metabolism in the current century).

In 2013 Chang et al. (85) connected the enhanced aerobic glycolysis in activated TCs to post-transcriptional control of cytokine secretion. Interestingly, the switch to glycolysis was not necessary for TC-proliferation and survival but for their ability to produce the effector cytokines IFN-γ and IL-2 (Figure 2) (85). Surprisingly, the glycolytic enzyme GAPDH was shown to bind and inhibit IFN-γ mRNA, making glycolytic enzymes an additional direct regulator of TC effector function (Figure 2) (85). As metabolic regulation of nucleic acids probably occurs also in other immune cells and other metabolic pathways, it is highly complicated to investigate and understand the underlying network. Here, the interaction of nucleic acids with cellular metabolism is an understudied field in immune metabolism that scientists should keep in mind to answer some of the currently remaining questions.

Moreover, Tamas et al. (86) demonstrated the activation of the TC receptor (TCR) to be both AMPK and CaMKK-dependent, both of which are crucial in regulating cellular energy homeostasis. Here, the elevation of intracellular Ca^2+^-levels in response to TCR/Ca^2+^-stimulation induced rapid AMPK activation, which depended on CaMKKs and the adapter molecules linker for the activation of TCs (LAT) and Scr homology 2 domain-containing leukocyte protein of 76 kDa (SLP76) (86).

In 2009, Delgoffe et al. (87) further showed mammalian target of rapamycin (mTOR) to regulate the development of effector and regulatory TC lineages. TCs lacking mTOR demonstrated decreased STAT activation and failed to differentiate into either Th1-, Th2-, or Th17-effector TCs (Figure 2) (87). mTOR-deficient TCs instead preferentially differentiated into Foxp3^+^ regulatory TCs (Treg) (87).

mTOR is a conserved serine/threonine protein kinase belonging to the PI3K family that senses and integrates diverse nutritional and environmental cues such as growth factors, cellular energy-, and stress levels (88). Recently, activation of the mTOR-pathway was also reported for TLR-ligands such as LPS, CPG, polyI:C, or flagellin (Figure 2) (88–93), suggesting mTOR to function as a master regulator with critical functions in immune cell development and function (88).

In 2011 Michalek et al. (94) showed that the differentiation of effector TCs and Tregs requires distinct metabolic programs: While effector Th1-, Th2-, and Th17-TCs are highly glycolytic expressing high levels of the glucose transporter GLUT1 (Figure 2), Tregs depended on lipid oxidation and expressed only low levels of GLUT1. Mechanistically, effector TCs require mTOR to promote glycolysis, while in Tregs, high levels of AMPK drive lipid oxidation (94). Interestingly, resting TCs were also shown to rely on lipid oxidation (95, 96). Therefore, mTOR is a major regulator of Th1-, Th2-, and Th17-differentiation, while mTOR-inhibition through AMPK promotes the differentiation of Tregs (94). These results, together with the fact that rapamycin was shown to prolong the lifespan of mice (97), suggest to investigate mTOR as a promising target to modulate the Th17/Treg cells axis, which plays a critical role in tolerance development [currently in focus for immune disorders (98)]. Here, we see the potential to reduce allergic inflammation, and with this, improve treatment outcomes.

As reported in 2007, the inhibition of ROS production by APCs resulted in reduced T cell stimulation and cytokine production during immune responses (99). On the other hand and in the combination with the previously described ROS generation, ROS were found 2010 to participate in the induction of Th2 responses, as they were shown to (I) inhibit the Th1-cytokine IL-12 and (II) recruit IL-4-producing basophiles (100). In contrast, reduced ROS levels, or treatment with antioxidants, were described to induce Th17 cell differentiation (31, 101).

In 2016, Buck et al. (102) furthermore showed the remodeling of mitochondrial structures to influence the metabolism of both effector and memory TCs. Here, mitochondrial morphology mainly influenced metabolic changes, especially the folds of the inner mitochondrial membrane, called cristae (102). Morphologically, mitochondria of memory TCs displayed a fused network, while effector TCs exhibited punctuated mitochondria (102). This fusion of mitochondria into tubular networks in memory TCs was shown to be optic atrophy type 1 (OPA1, controlling the mitochondrial inner membrane fusion events)-dependent and led to association of the ETC complex which favors FAO and OxPhos (102). In contrast, in effector TCs, the generation of fragmented mitochondria expanded cristae favoring aerobic glycolysis (102). The goal of future research should be to investigate, whether T cells forced to rely on a FAO-dominated metabolic phenotype, for example, have a regulatory phenotype with Th2-suppressive capacity. Currently, the effect of FA, FAO, and the resulting generation of modulating metabolic intermediates are under discussion to improve transplantation approaches by modulating Treg cell functions (103). One approach could be to modulate glycolysis-based metabolism in effector T cells toward FAO metabolism-based metabolism by targeted inhibition of glycolysis, e.g., *ex vivo* via 2-DG, thereby promoting Treg differentiation.

These pioneering studies showed, that effector TCs rely on mTOR-activation (87), glycolysis (84), and the processing of glucose during their activation (85), while both resting memory TCs and Tregs mainly rely on FAO (94–96). During both processes different metabolites accumulate, which not only interact intracellularly, but can also directly act on other immune cells. For example lactate was recently shown to inhibit LPS-induced mast cell function by inhibiting glycolysis (104) leading to an acidic environment, which was shown to block the activation of T cells *in vitro* (105) and have a direct effect on microbiota [reviewed in (106)].

### Activated T Cells Are Fueled by Glutamine

Carr et al. (107) showed in 2010, that the activation of TCs requires CD28-dependent uptake of the amino acid glutamine (Figure 2). Here, TC-activation resulted in both enhanced expression of glutamine transporters and enhanced activation of enzymes involved in glutamine metabolism (107). Via glutamate that is further converted to α-ketoglutarate and metabolized in the Krebs cycle, glutamine can act as an energy source (Figure 2) (7, 107). Concordantly, depletion of glutamine inhibited T cell proliferation and cytokine production (107). Both increased glutamine import and -metabolism were shown to be highly dependent on extracellular regulated kinase (ERK)-signaling (Figure 2) (107). Additionally, ERK signaling is responsible for changes in glucose metabolism via TCR/CD28-signaling, suggesting ERK to be another important regulator of T cell metabolism (107).

In line with these results, Wang et al. (108) published 2011 that the transcription factor Myc reprograms the metabolism of activated TCs toward a combination of FAO, pyruvate oxidation (OxPhos), aerobic glycolysis, the pentose phosphate pathway, and glutaminolysis associated with the synthesis of macromolecules (lipids, amino acids, and nucleotides), proliferation, and cell growth. Restriction of glutamine, but not glucose, decreased lipid- and protein biosynthesis (108), underlining the connection between immune response and nutrition catabolism in immune cells. These results also highlight how the diversity of nutrients used to generate effector functions of activated T cells and pharmacological inhibition of nutrient catabolism pathways may affect the outcome of allergy therapies. Therefore, further investigation of immune metabolism during AIT is required to understand the underlying metabolic changes more both in detail and over the time of treatment.

## Relevance of Immune Metabolism for Allergic Diseases

**Figure 3:**
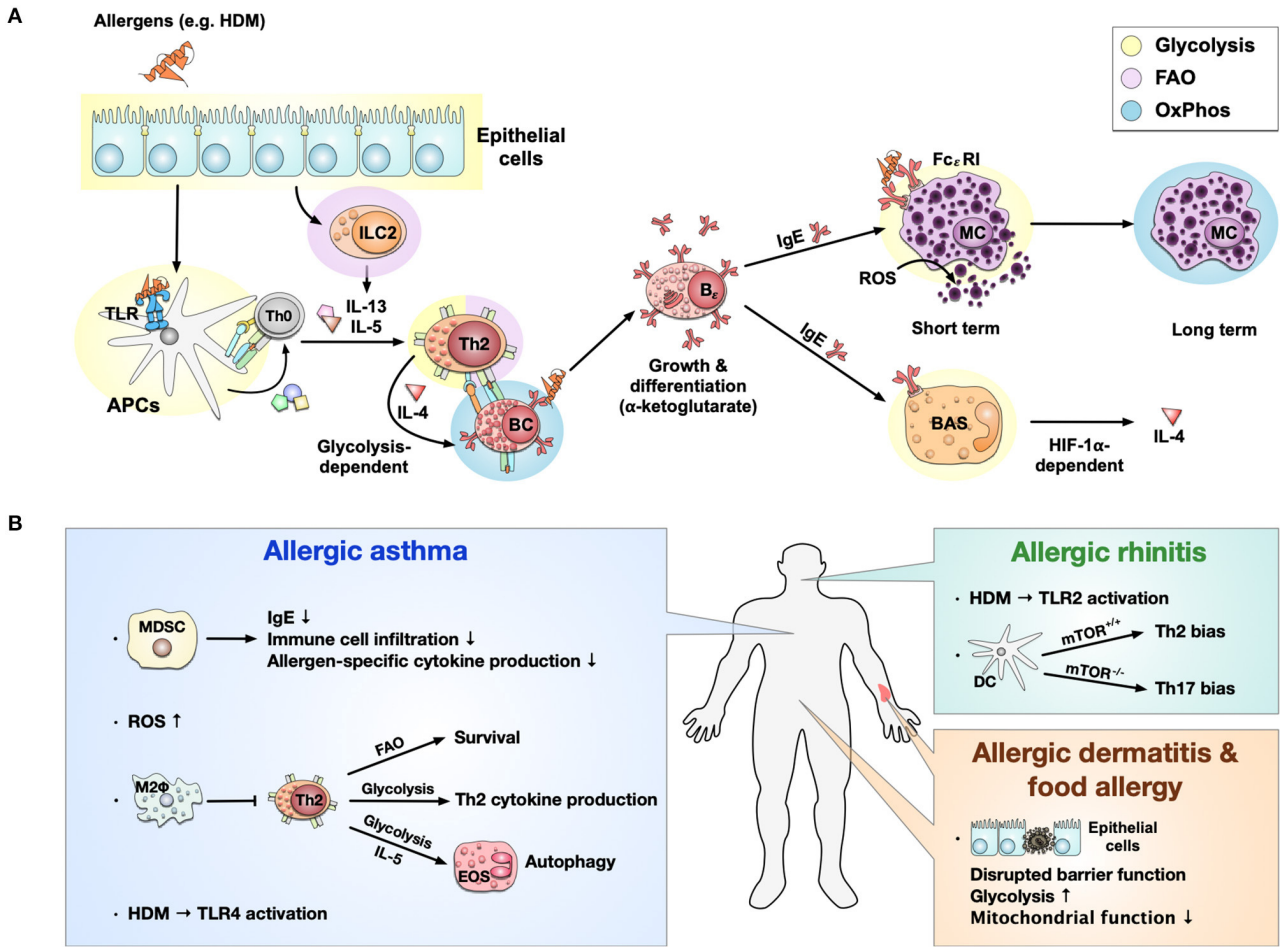
Immune metabolism in allergies. Immune metabolism in allergic sensitization and pathology **(A)**. Allergens, e.g., derived from house dust mites (HDM), activate epithelial cells and induce a metabolic shift toward glycolysis. Epithelial cells further activate ILC2s and induce FAO-dependent production of the Th2 cytokines IL-5 and IL-13. Some allergens can also bind to TLRs, causing a switch from OxPhos to glycolysis in APCs. Activated ILC2s and APCs further induce the differentiation of naïve T cells into Th2 cells which produce IL-4 in a glycolysis-dependent manner. The activation and differentiation of B cells into IgE-producing plasma cells after IL-4 stimulation is dependent on the Krebs cycle-derived metabolite α-ketoglutarate. The produced IgE binds to the high-affinity FCεRI on mast cells, where cross-linking of IgE leads to a short-term glycolytic phenotype. In contrast, long-term mast cell activation is dependent on OxPhos. Additionally, high concentrations of ROS can directly activate mast cells to release histamine and serotonin. On the other hand, IgE can also bind to basophils, leading to a HIF-1α-dependent activation of glycolysis and subsequent IL-4 production. Immune metabolic changes observed in allergic asthma, rhinitis, dermatitis, and food allergy **(B)**. In allergic asthma, MDSCs have been shown to inhibit allergic asthma by reducing IgE production, immune cell infiltration, and allergen-specific cytokine production. At the same time, high levels of reactive oxygen species (ROS) were found. OxPhos-based anti-inflammatory M2 macrophages may locally inhibit Th2 responses in allergic asthma. Furthermore, for Th2 cells, FAO-dependency could be identified to be essential for survival, while glycolysis was essential for Th2 cytokine production. IL-5 can activate eosinophils, shift their metabolism toward a glycolytic phenotype, and affect their effector functions like autophagy in asthma. Allergic asthma induced by HDM was shown to be TLR4-dependent; while HDM-induced allergic rhinitis was driven by TLR2 activation. In allergic rhinitis, mTOR-deficiency in CD11b^+^ DCs induced Th17- instead of Th2-biased immune responses. While little is known about immune metabolism in allergic dermatitis and food allergy, they are both connected to a disrupted barrier function of epithelial cells characterized by a predominantly glycolytic phenotype and mitochondrial dysfunction. TLR, “Toll”-like receptor; APC, antigen presenting cell; MC, mast cell; BAS, basophil; ROS, reactive oxygen species; HIF-1α, hypoxia inducible factor 1 alpha; MHC, major histocompatibility complex; mTOR, mammalian target of rapamycin; FAO, fatty acid oxidation; HDM, house dust mite; IgE, Immunoglobulin E; B_ϵ_, IgE-producing B cell; BC, B cell; TC, T cell; OxPhos, oxidative phosphorylation; MDSC, Myeloid-derived suppressor cell; Fc_ε_RI, fragment crystallizable region epsilon receptor I; M2Φ, M2-macrophages; EOS, eosinophils.

As described above, metabolic changes in immune cells are closely related to their effector functions. Therefore, cells involved in allergies also display distinct metabolic profiles (see Figure 3A). Here we provide a short overview, but recently summarized these findings in more detail (36).

During allergic sensitization, epithelial cells are the first line in contact with allergens. Currently, most of the available studies focus on air way epithelial cells in asthma (109–113), while only a few reports described intestinal epithelial cells in food allergy (114) and atopic dermatitis (115). All types of epithelial cells shift their metabolism toward glycolysis during allergic inflammation, accompanied by mitochondrial dysfunction, resulting in local inflammation and disrupted barrier integrity (Figures 3A,B) (109–115).

Subsequently, allergens can cross disrupted barriers and are taken up by APCs like macrophages and DCs. As mentioned above, activated APCs display a strongly glycolytic phenotype accompanied by a disrupted Krebs cycle (Figures 2, 3A). A recent study in 2021 also demonstrated that the Krebs cycle enzyme aconitase decarboxylase and the enzyme product itaconate both regulated DC activation and were critical for type 2 allergic asthma development (116). Another point of connection between metabolism and the regulatory role of DCs in allergies is the mTOR complex. Sinclair et al. (117) reported that when mice were challenged with HDM intranasal, mTOR-deficient CD11b^+^ DCs induced a neutrophilic Th17 response rather than Th2 inflammation (Figure 3A). Compared to DCs, the role of macrophage metabolism in allergic diseases is more controversial. Previous studies indicated that M2 anti-inflammatory macrophages (OxPhos phenotype) play an important role in asthma and food allergy (118–121). Besides, M1 pro-inflammatory macrophages (glycolytic phenotype) (122, 123) are also involved in asthma reactions. Interestingly, a study in a mouse model of 2,4-dinitrofluorobenzene-induced contact hypersensitivity demonstrated that M2 macrophages protected from local Th2 responses (124). These findings demonstrated that APCs might show different metabolic phenotypes in different allergic responses and further affect allergic reactions.

In allergies, epithelial cells trigger innate-like lymphocytes type II cells (ILC2s) to secrete Th2-promoting cytokines, favoring the differentiation of naive T cells into Th2 cells. Several reports indicated FAO to be critical for ILC2 function, including ILC2 accumulation, IL-5, and IL-13 secretion (Figure 3A) (125–127). For allergen-specific Th2 cells, glycolysis was shown to be important for Th2 cytokine production (128, 129), and FAO was involved in Th2 cells survival (130, 131) in airway allergic responses (Figure 3B).

Th2 cytokines (e.g., IL-4) further induce isotype switching of allergen-specific B cells to IgE-producing plasma cells. Studies showed that IL-4-stimulated B cells enhance mitochondrial metabolism by generating Krebs cycle intermediates (e.g., α-ketoglutarate), promoting B cell growth and differentiation (Figure 3A) (132, 133).

Upon crosslinking of IgE antibodies bound to the FcεRI on the surface of mast cells, these cells become activated, degranulate, and trigger the allergic inflammation cascade. Interestingly, short-term activation of mast cells mainly increased glycolysis by FcεRI-mediated cell activation (134), while in the long-term mast cells displayed a switch toward a more OxPhos phenotype (Figure 3A) (135, 136). In contrast, current studies on either eosinophil- or basophil-metabolism during allergic reactions are limited. Few reports indicated, that upon IL-5-stimulation, eosinophils display a glycolytic phenotype, affecting effector functions like autophagy in asthma (137, 138). HIF-1α plays an important role in IgE-mediated basophil activation and further mediates cell adaption to hypoxic conditions by promoting a shift toward a glycolytic phenotype that provides ATP for IL-4 (and vascular endothelial growth factor) production (Figure 3A) (139, 140).

Taken together, all major cell types involved in allergic inflammation display metabolic changes with epithelial cells, DC, M1-macrophages, eosinophils, and basophils shifting toward glycolysis, ILC2s mainly relying on FAO, and Th2 cells depending on both glycolysis and FAO. Acutely activated mast cells display a glycolytic phenotype while in the long-term changing to OxPhos. Finally, M2-macrophages and IL-4-stimulated B cells present an OxPhos-dominated metabolic phenotype (summarized in Figure 3A). These findings have the potential to both improve our understanding of allergic pathology and establish new therapy-targets in the future.

Since immune metabolism is a newly formed research field, studies on especially allergic diseases are limited (summarized in Figure 3B). Current reports on metabolic changes in different cell types are mainly based on *ex vivo* cell culture or *in vivo* asthmatic models. Clinical samples analyzed so far were also mainly obtained from asthma patients. Therefore, it would be interesting to know if cells involved in different allergic responses (e.g., food allergy or atopic dermatitis) also show different metabolic profiles. A recent clinical study hinted at this possibility by analyzing blood metabolomics profiles from children with either food allergy alone, asthma alone, or both (141). Interestingly, when compared to both normal subjects and asthmatic patients, food allergic subjects had a specific metabolomic signature with a significant decrease in sphingolipid metabolism (141). Besides, patients with both food allergy and asthma had a metabolomics profile that was more similar to food allergy than to asthma (141).

## Conclusion

We have come a long way in understanding immune cell metabolism since the pioneering findings of Otto Warburg. Modern analyses methods, knockout technology, and the advent of OMICS technologies have allowed us to dissect the contribution of single-cell types, genes, and metabolic pathways to the initiation, maintenance, and resolution of immune responses. As detailed in this paper, this progress has already improved our understanding of allergic pathology and may lead to novel target molecules with the potential to improve the treatment of allergic diseases.

## Author Contributions

All authors listed have made a substantial, direct, and intellectual contribution to the work and approved it for publication.

## Funding

This work was in part funded by the budget of the Paul-Ehrlich-Institut, Langen, Germany. AG was funded by the German Research Foundation (DFG SCHU2951/4). Y-JL was funded by the German Research Foundation (DFG SCHE637/4).

## Conflict of Interest

The authors declare that the research was conducted in the absence of any commercial or financial relationships that could be construed as a potential conflict of interest.

## Publisher's Note

All claims expressed in this article are solely those of the authors and do not necessarily represent those of their affiliated organizations, or those of the publisher, the editors and the reviewers. Any product that may be evaluated in this article, or claim that may be made by its manufacturer, is not guaranteed or endorsed by the publisher.

## Glossary

4-OI: 4-octyl itaconate
Akt: protein kinase B
AMPK: adenosine monophosphate (AMP)–activated protein kinase
APCs: antigen presenting cells
CaMKKβ: calcium/calmodulin-dependent protein kinase kinase β
DC: dendritic cell
ECAR: extracellular acidification rate
ECSIT: evolutionarily conserved signaling intermediate in Toll pathways
EIF2AK2: eukaryotic translation initiation factor 2 alpha kinase 2
ERK: extracellular regulated kinase
ETC: electron transport chain
FAO: fatty acid oxidation
GAPDH: glyceraldehyde-3-phosphate dehydrogenase
GC: germinal centers
G-CSF: granulocyte colony-stimulating factor
GDH: glutamate dehydrogenase
GLUT1: glucose transporter 1
GM-CSF: granulocyte/macrophage colony-stimulating factor
GSH: glutathione
HDM: house dust mite
HIF-1α: hypoxia-inducible factor 1α
I-152: N-(N-acetyl-l-cysteinyl)-S-acetylcysteamine
IDH: isocitrate dehydrogenase
IDO: indoleamine 2, 3-dioxygenase
ILC2s: innate-like lymphocytes type 2 cells
IRG-1: immune-responsive gene 1
LAT: linker for the activation of T cells
LKB1: liver kinase B1
LPS: lipopolysaccharide
MHC: major histocompatibility complex
mTOR: mammalian target of rapamycin
M-CSF: macrophage colony-stimulating factor
M/G-MDSC: monocytic/ granulocytic myeloid derived suppressor cells
NEK7: NIMA-related kinase 7
NOS: nitric oxide species
NOX(-2): NADPH oxidase (2)
OPA1: optic atrophy type 1
OxPhos: oxidative phosphorylation
PAMPs: pathogen-associated molecular patterns
PDH: pyruvate dehydrogenase
PI3K: phosphatidyl inositol 3-kinase
PKM2: pyruvate kinase isozyme M2
ROS: reactive oxygen species
SDH: succinate dehydrogenase
SIRT5: Sirtuin 5
SLP76: Scr homology 2 domain-containing leukocyte protein of 76 kDa
TCs: T cells
TLR: “Toll”-like receptor
TRAF6: tumor necrosis factor receptor-associated factor 6.
